# Immunological response among patients with undetectable viral load followed for 5 years

**DOI:** 10.1186/1758-2652-13-S4-P18

**Published:** 2010-11-08

**Authors:** C Piñeiro, AS Santos, S Cardoso, J Nuak, R Lucas, A Ferreira, S Xerinda, M Tavares, R Marques, A Sarmento

**Affiliations:** 1 Hospital São João, Infectious Diseases, Porto, Portugal; 2 University of Porto Medical School, Department of Hygiene and Epidemiology, Porto, Portugal

## Purpose of the study

to assess the immunological response at 5 years in HIV-1 infected patients under ART and with persistently undetectable plasma viral load.

## Methods

of 1450 patients currently on ART, 504 HIV-1 infected patients with persistently undetectable viral load for 5 or more years (mean = 8±2 years) were selected. Results of plasma viral load (performed by PCR RNA-HIV, Amplicor 1.5®) and CD4 count of all patients obtained every 3 or 6 months were abstracted. The distribution of 5-year CD4 count was described using medians and compared between classes of clinical and demographic variables using Wilcoxon's or Kruskal-Wallis' test. Variation in CD4 count over 5 years was described using means and compared between classes using Student's t test or ANOVA. In order to quantify the associations between independent variables and CD4 count variation, linear regression coefficients and respective 95% confidence intervals (95% CI) were calculated, and adjusted for sex, naïve status, transmission mode, baseline viral load and age at viral load suppression.

## Summary of results

In crude analysis, 5-year CD4 count (per mm^3^) was significantly higher in women (medians: 656 vs. 573, p=0.006), in younger patients (<30 years-old: 656 vs. 526 in those over 49), in non-AIDS patients (637 vs. 513.5 in AIDS patients, p<0.001) and in those with the highest baseline CD4 count (>350: 826 vs. 459 in those under 100; p<0.001). Mean CD4 variation was greater in women (467.5 vs. 401.3, p=0.022), in naïve patients (437.9 vs. 361.8, p=0.006), in those with the highest baseline viral load (>750000 copies: 490.3 vs. 271.0 in those under 10000 copies) and correspondingly in patients with the lowest baseline CD4 count (<100: 469.7 vs. 359.5 in those over 350). Patients on NNRTI had larger improvement than those on PI (436.2 vs. 403.0) but no significant differences in CD4 count were found after 5 years. In multivariate analysis, mean CD4 count improvement remained significantly higher in women and lower in older patients. Improvement was also directly associated with baseline viral load.

## Conclusions

Even though patients with the lowest CD4 count at baseline had greater immunological improvement over 5 years, average CD4 count remained lower than in those whose baseline count was higher. Importantly, the gap in immunological response (regarding final CD4 count and mean improvement) widened over the follow-up period between women and men and between younger and older patients.

